# Treatment Using Both Voclosporin and Belimumab in Four Patients With Lupus Nephritis

**DOI:** 10.7759/cureus.38848

**Published:** 2023-05-10

**Authors:** Rebecca Baum, Duruvu Geetha, Ayotola Fatola, Homa Timlin

**Affiliations:** 1 Rheumatology, Johns Hopkins University School of Medicine, Baltimore, USA; 2 Nephrology, Johns Hopkins University School of Medicine, Baltimore, USA; 3 Internal Medicine, Johns Hopkins University School of Medicine, Baltimore, USA

**Keywords:** proteinuria, lupus nephritis flare, systemic lupus erythematosis, positive ana, sle and lupus nephritis

## Abstract

Nearly 50% of patients with systemic lupus erythematosus (SLE) will develop lupus nephritis (LN). Current treatment regimens for LN are suboptimal as the majority of patients fail to achieve complete renal response after several months of treatment and there are high rates of relapse. We report outcomes in four LN patients who were treated with both voclosporin and belimumab. These patients had no serious infections, and we were able to taper glucocorticoids and reduce proteinuria.

## Introduction

Lupus nephritis (LN) and a lack of response to therapy are associated with an increased risk of progressive kidney disease [1]. Recently, two medications - belimumab [2] and voclosporin [3] - have been approved by the FDA as add-on therapy for LN. However, there is a lack of studies assessing the efficacy, tolerability, and safety of combination treatment involving voclosporin and belimumab. We report the outcomes in four African American female patients with biopsy-proven LN and severe lupus who were treated with both voclosporin and belimumab.

## Case presentation

Case 1

A 33-year-old woman with LN class III and V was started on mycophenolate mofetil (MMF), glucocorticoids, and hydroxychloroquine. Due to intolerance to MMF, mycophenolic acid, and tacrolimus, she was eventually treated with azathioprine, belimumab, hydroxychloroquine, and prednisone. Multiple attempts to bring her prednisone dosage below 7.5 mg daily resulted in flares including recurrence of proteinuria, oral ulcers, and discoid rash.

Within three weeks of starting voclosporin, she noted the resolution of oral ulcers and reduced discoid scalp flares. She tapered off prednisone and azathioprine. She has been on voclosporin, belimumab, and hydroxychloroquine over a period of 14 months with no further flares. Proteinuria has also resolved.

Case 2

A 34-year-old woman with systemic lupus erythematosus (SLE) developed proteinuria and was found to have LN class II and V. MMF was added to her regimen of hydroxychloroquine and prednisone. Her urine protein-creatinine ratio (UPCR) remained elevated at 2500 mg/g after three months of MMF therapy and hence voclosporin was added. Within four weeks, UPCR decreased to 800 mg/g. Three months later, she continued to have ongoing disease activity as manifested by synovitis, rash, leukopenia, nasal ulcers, and continued proteinuria (UPCR >500 mg/g) and hence belimumab was added.

The patient was on high-dose MMF, voclosporin, belimumab, and hydroxychloroquine for 12 months. MMF was discontinued one month ago. Prednisone was tapered to 2.5 mg daily. Her eGFR remains stable with complete resolution of proteinuria.

Case 3

A 28-year-old woman with SLE and class V LN developed new proteinuria (UPCR 6180 mg/g). Her creatinine increased from a baseline of 0.7 mg/dl to 1.6 mg/dl, and a kidney biopsy revealed LN class III/IV. She had previously been taking MMF but did not take it consistently due to diarrhea. She declined cyclophosphamide. She was started on Myfortic, glucocorticoids, and tacrolimus. Six months later, it was found that her proteinuria persisted (UPCR 1460 mg/g). Therefore, belimumab was added, and six months after that tacrolimus was switched to voclosporin.

The patient remains on Myfortic, voclosporin, belimumab, and hydroxychloroquine, and has tapered off prednisone for the past five months. She has not had any further incidences of synovitis or oral ulcers, and there has been an improvement in proteinuria (UPCR decreased below 500 mg/g) and creatinine (decreased to 1.2 mg/dl).

Case 4

A 33-year-old woman with SLE and LN class III was treated with glucocorticoids, MMF, tacrolimus, and hydroxychloroquine. Three years later, she developed leukopenia after a coronavirus disease 2019 (COVID-19) infection; MMF was held and she developed proteinuria. At this time, tacrolimus 2 mg twice daily was switched to voclosporin 23.7 mg twice daily. Eight months later, belimumab was added for arthritis and the voclosporin dose was reduced to 15.8 mg twice daily. She has been on voclosporin, belimumab, and Plaquenil for the last nine months. On this regimen, her kidney function has remained stable, and proteinuria resolved. She has also been able to taper off prednisone (Table 1).

**Table 1:** Outcomes in four patients with biopsy-proven LN and severe lupus who were treated with both voclosporin and belimumab LN: lupus nephritis; voc.: voclosporin; bel.: belimumab; nr: normal range; HCQ: hydroxychloroquine; bid: twice per day; q: every; qhs: at night; qam: in the morning; UPCR: urine protein-creatinine ratio Mild COVID-19: mild respiratory symptoms that resolved within one week. Not hospitalized

Case	Age (years)	Gender	Extra-renal clinical features	Lab results	LN class	Current medications	Months on voc./bel.	Treatment response	Infections
1	33	Female	Oral ulcers, arthritis, alopecia, discoid lupus, pleurisy	ANA >1:640 speckled, dsDNA >1:640 (nr <10 U), Smith >693 (nr <20 U), RNP >643 (nr <20 U), Ro60 >1374 (nr <20 U), leukopenia, thrombocytopenia, low C3	III, V	Voc. 15.8 mg bid, Bel. 200 mg q7 days, HCQ 400 mg daily	14	Reduced oral ulcers, reduced discoid flares, tapered off prednisone, tapered off azathioprine, proteinuria resolved (UPCR down from 290 to 60 mg/g)	Mild COVID-19
2	34	Female	Oral ulcers, arthritis, alopecia, chilblains	ANA >1:640 speckled, SSA >8 (nr 0-0.9), low C3, leukopenia	II, V	Voc. 23.7 mg bid, Bel. 200 mg q7 days, HCQ 400 mg daily, prednisone 2.5 mg daily	12	Prednisone tapered, mycophenolate discontinued, proteinuria resolved (UPCR down from >500 to 65 mg/g)	None
3	28	Female	Oral ulcers, arthritis, alopecia, bullous lupus	ANA 1:1280 speckled, dsDNA >1:640 (nr <10 U), Smith >693 (nr <20 U), RNP >643 (nr <20 U), low C3	III, IV, V	Myfortic 720 mg bid, Voc. 23.7 mg qam and 15.8 mg qhs, Bel. 200 mg q7 days, HCQ 300 mg daily	8	Synovitis resolved, prednisone discontinued, proteinuria subsided (UPCR down from 1460 to <500 mg/g)	Mild COVID-19
4	33	Female	Oral ulcers, arthritis, alopecia	ANA >1:640 speckled, dsDNA >1:640 (nr <10 U), Ro52 >1685 (nr <20 U), low C3	III	Voc. 15.8 mg bid, Bel. 200 mg q7 days, HCQ 400 mg daily	9	Tapered off prednisone, proteinuria resolved (UPCR down from 520 to 107 mg/g)	None

## Discussion

There exists an unmet need for the treatment of LN. Of note, 20% of patients with LN will progress to end-stage renal disease within 10 years [4]. Ginzler et al. found that only 23% of patients with LN on MMF were in complete renal remission after three months of therapy, a remarkably low percentage [5]. The recent BLISS LN study [2] showed superior outcomes with the addition of belimumab in patients with LN; however, the response rate was slow. GFR protection was shown as early as four months and it was maintained at two years. Proteinuria was reduced as rapidly as within two weeks in the voclosporin AURORA [3] trial. Notably, around 60% of patients in the BLISS LN and AURORA trials did not achieve complete renal response, highlighting the need for alternative approaches toward LN treatment.

Many questions remain unanswered regarding the management of LN, such as whether combination therapy with voclosporin and belimumab could be used safely and replace steroids and mycophenolate in LN treatment.

## Conclusions

We described the cases of four patients with LN treated with the combination therapy of voclosporin and belimumab for 8-14 months, without evidence of any serious infections or adverse side effects thus far. Moreover, they managed to taper off glucocorticoids with a reduction of proteinuria. Future clinical trials should formally assess the safety and efficacy of the treatment of LN with the combination of voclosporin and belimumab.
